# Imaging features of Takayasu arteritis in a young male

**DOI:** 10.1093/ehjimp/qyaf076

**Published:** 2025-06-04

**Authors:** Maria Ana Estevens, Ana Coutinho Santos, Duarte Saraiva Martins, Carla Saraiva

**Affiliations:** Paediatric Cardiology Department, Hospital de Santa Cruz, Unidade Local de Saúde Lisboa Ocidental, 2790-134 Carnaxide, Portugal; Radiology Department, Hospital de Santa Cruz, Unidade Local de Saúde Lisboa Ocidental, Carnaxide, Portugal Ocidental, Carnaxide, Portugal; Paediatric Cardiology Department, Hospital de Santa Cruz, Unidade Local de Saúde Lisboa Ocidental, 2790-134 Carnaxide, Portugal; Radiology Department, Hospital de Santa Cruz, Unidade Local de Saúde Lisboa Ocidental, Carnaxide, Portugal Ocidental, Carnaxide, Portugal

**Keywords:** Takayasu arteritis, aortitis syndrome, vasculitis, multi-segment vascular involvement, CT angiography, MRI vascular imaging

## Clinical message

We present a 17-year-old male with no prior medical history who developed fatigue, unintentional weight loss, night sweats, and recurrent fever. A thoracoabdominal non-gated CT scan revealed a fusiform aneurysm of the ascending aorta and transverse aortic arch, with circumferential thickening of the aortic wall and supra-aortic trunks. MR and ECG-gated CT angiography performed a week later confirmed ascending aorta aneurysm (52 mm), vascular wall thickening, and T2 hyperintensity suggestive of wall oedema, without dissection. The carotid arteries were also involved, raising suspicion of multi-segment vasculitis. Given the extensive vascular involvement, the differential diagnosis included autoimmune, infectious, and genetic causes. Infectious aetiologies such as syphilitic aortitis, tuberculosis, mycotic aneurysms, and HIV-related vasculopathy were excluded through serological and microbiological testing. Additionally, no clinical or imaging findings suggested Marfan, Loeys–Dietz, or Ehlers–Danlos syndromes, effectively ruling out genetic connective tissue disorders. Among autoimmune vasculitis, Takayasu arteritis was the most compatible diagnosis due to its predilection for young individuals and characteristic involvement of large and medium sized vessels. Other vasculitis were deemed less likely. With infectious and genetic causes excluded, and in the presence of compatible imaging findings and systemic symptoms, Takayasu arteritis emerged as the most probable diagnosis, guiding treatment and follow-up. The patient is still awaiting ¹⁸F-FDG PET, which has limited availability at our centre. One month later, when immunosuppressive therapy was tapered, the patient developed pallor and a diminished pulse in the left upper limb and ESR raised, further reinforcing the diagnosis. This case underscores the importance of a structured approach in evaluating young patients with systemic symptoms and vascular abnormalities.

**Consent:** Written informed consent was obtained from the patient for the publication of this study and any accompanying images. The authors confirm that this research was conducted in accordance with the ethical standards outlined by the Committee on Publication Ethics (COPE) and the Declaration of Helsinki. All necessary measures were taken to protect patient confidentiality and ensure compliance with institutional and international ethical guidelines.

**Funding:** None.

**Data availability:** All relevant data are included within the manuscript. This study is based on clinical case data, and no original or third-party datasets were used. In accordance with the policies of *EHJ – Cardiovascular Imaging*, the underlying deidentified data can be made available to the journal upon request for inspection and verification during the peer review process.

**Author contributions:** M.A.E.: Writing—original draft preparation. A.C.S.: Writing—preparation. D.S.M.: Writing—preparation. C.S.: Supervision, validation.

## Lead author biography

Maria Ana Estevens, MD, is a final-year fellow in Paediatric Cardiology at Hospital de Santa Cruz, Unidade Local de Saúde de Lisboa Ocidental (ULSLO), Portugal. Her main clinical and research interests include congenital heart disease, foetal cardiology, and long-term outcomes in paediatric and adolescent patients. She has contributed to retrospective and prospective studies on Tetralogy of Fallot and Fontan circulation, and has presented her work at both national and international scientific meetings. Dr Estevens is currently completing an observership in Paediatric Cardiology at Boston Children’s Hospital to further develop her expertise in advanced cardiovascular imaging.

